# G-protein-coupled receptor 81 promotes a malignant phenotype in breast cancer through angiogenic factor secretion

**DOI:** 10.18632/oncotarget.12286

**Published:** 2016-09-27

**Authors:** Yu Jin Lee, Kyeong Jin Shin, Soo-Ah Park, Kyeong Su Park, Seorim Park, Kyun Heo, Young-Kyo Seo, Dong-Young Noh, Sung Ho Ryu, Pann-Ghill Suh

**Affiliations:** ^1^ School of Life Sciences, Ulsan National Institute of Science and Technology (UNIST), Ulsan, Republic of Korea; ^2^ New Experimental Therapeutics Branch, Division of Convergence Technology, National Cancer Center, Goyang-si, Republic of Korea; ^3^ Department of Surgery, Seoul National University, College of Medicine, Seoul, Republic of Korea; ^4^ Department of Life Science, Pohang University of Science and Technology (POSTECH), San31, Hyoja Dong, Pohang, Republic of Korea

**Keywords:** GPR81, breast cancer, amphiregulin, angiogenesis, Akt

## Abstract

G-protein-coupled receptor 81 (GPR81) functions as a receptor for lactate and plays an important role in the regulation of anti-lipolytic effects in adipocytes. However, to data, a role for GPR81 in the tumor microenvironment has not been clearly defined. Here, GPR81 expression in breast cancer patients and several breast cancer cell lines was significantly increased compared with normal mammary tissues and cells. GPR81 knockdown resulted in impaired breast cancer growth and led to apoptosis both *in vitro* and *in vivo*. Furthermore, the inhibition of GPR81 signaling suppressed angiogenesis through a phosphoinositide 3-OH kinase (PI3K)/Akt-cAMP response element binding protein (CREB) pathway, which led to decreased production of the pro-angiogenic mediator amphiregulin (AREG). Overall, these findings identify GPR81 as a tumor-promoting receptor in breast cancer progression and suggest a novel mechanism that regulates GPR81-dependent activation of the PI3K/Akt signaling axis in tumor microenvironment.

## INTRODUCTION

Breast cancer is the leading cause of cancer-related mortality in females worldwide [1]. Our understanding of the molecular mechanisms of breast cancer has improved in the previous two decades; however, the prognosis and treatment of breast cancer, particularly in advanced cases, has not been significantly improved [2, 3]. Breast cancer is a heterogeneous disease defined by different activating mutations, epigenetic modifications and aberrant signaling pathways. Thus, a more detailed understanding of the molecular mechanisms underlying the breast cancer progression will provide new insight into individual treatment of breast cancer.

G-protein-coupled receptors (GPCRs) comprise seven-transmembrane proteins that regulate important physiological processes, through the coordinated action of their signaling pathways [4–6]. Altered gene expression and dysregulated GPCR signaling have been recognized as hallmarks of cancer [7, 8]. Abnormal expression of specific GPCRs on cell membranes stimulates the continual unregulated proliferation and triggers intracellular signal transductions that ultimately lead to the growth of cancer cells, induction of angiogenesis and metastasis. Approximately 50% of marketed pharmaceuticals target human GPCRs or their signaling pathways; however, a limited number of these receptors are used as cancer therapeutic targets [9, 10]. A tremendous amount of efforts have been made to so far aiming at exploiting therapeutic applications of the remaining family members, including more than 140 orphan GPCRs whose endogenous ligands or functions have yet to be unidentified [11].

Recently, an increasing number of orphan GPCRs have been demonstrated to be activated by metabolic intermediates or energy substrates [12, 13]. In particular, the GPR81 family of receptors consists of three members that are primarily expressed in adipocytes (GPR109a, GPR109b and GPR81), and activation by their respective agonists (3-hyfroxybutyrate, 3-hydroxyoctanoate, and lactate) inhibits adipocyte lipolysis [14]. It has been proposed to have an important role in metabolic disorders, such as dyslipidemia and type 2 diabetes [15, 16]. In tumor microenvironment, malignant cancer cells with enhanced glucose uptake export lactate as a by-product of glycolysis. However, the expression of GPR81 and its role in breast cancer progression have not been previously reported in the context of the tumor microenvironment.

Here, we describe the role of GPR81 in the pathogenesis of human breast cancer. We provide evidence that GPR81 promotes proliferation via the inhibition of apoptosis and stimulates the secretion of several angiogenic factors in a PI3K/Akt-CREB signaling pathway-dependent manner. Taken together, our findings suggest that GPR81 functions to promote cancer cell survival and angiogenesis and represents a potential target for breast cancer treatment.

## RESULTS

### GPR81 is aberrantly expressed in breast cancer

**Figure 1:**
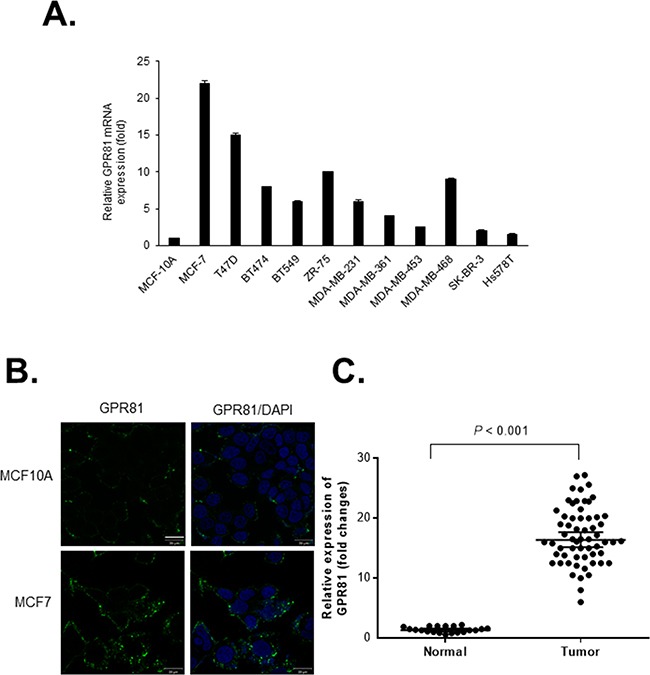
GPR81 is highly expressed in breast cancer **A.** Quantitative PCR (qPCR) analyses of GPR81 mRNA expression in eleven human breast cancer cell lines and immortalized normal human mammary epithelial cells (MCF10A). The relative mRNA expression of GPR81 is presented as the mean±SEM; n≥3. **B.** GPR81 immunofluorescence staining of MCF10A or MCF7 cells (Green). Nuclei were counterstained with DAPI. The scale bar represents 20 μm. **C.** Relative mRNA expression of GPR81 in breast cancer (n=60) versus normal (n=20) tissues (unpaired two-tailed *t*-test).

To determine GPR81 expression in breast cancer, we analyzed the GPR81 mRNA levels in various breast cancer cell lines. GPR81 mRNA was detected in 8 of 11 (72%) breast cancer cells compared with a normal mammary epithelial cell line, MCF10A (Figure 1A). Among these cancer cell lines, MCF7 cells, which highly expressed GPR81 (a 22-fold increase compared with MCF10A cells), were further investigated. Moreover, the GPR81 mRNA levels correlated with the protein levels, as indicated by immunofluorescence. Increased GPR81 protein levels were identified in MCF7 cells, while decreased levels were found in MCF10A cells (Figure 1B). For clinical relevance, we examined the GPR81 expression in archived breast cancer tissues and normal breast tissues using quantitative polymerase chain reaction (qPCR). As shown in Figure 1C, the GPR81 expression in cancer tissues was significantly increased compared with the adjacent noncancerous tissues (*P*<0.001). In addition, an analysis of a large cancer dataset by the cBioPortal [17, 18] indicated that breast cancers showed the greatest number of genomic copies of GPR81 compared with other cancer types (Supplementary Figure S1A). Interestingly, increased GPR81 expression was correlated with the estrogen receptor (ER)-positive status of breast cancer patients (Supplementary Figure S1B), which suggests that GPR81 may comprise an important regulator or prognostic marker of breast cancer.

### GPR81 promotes breast cancer cell proliferation and inhibits apoptosis *in vitro*

**Figure 2:**
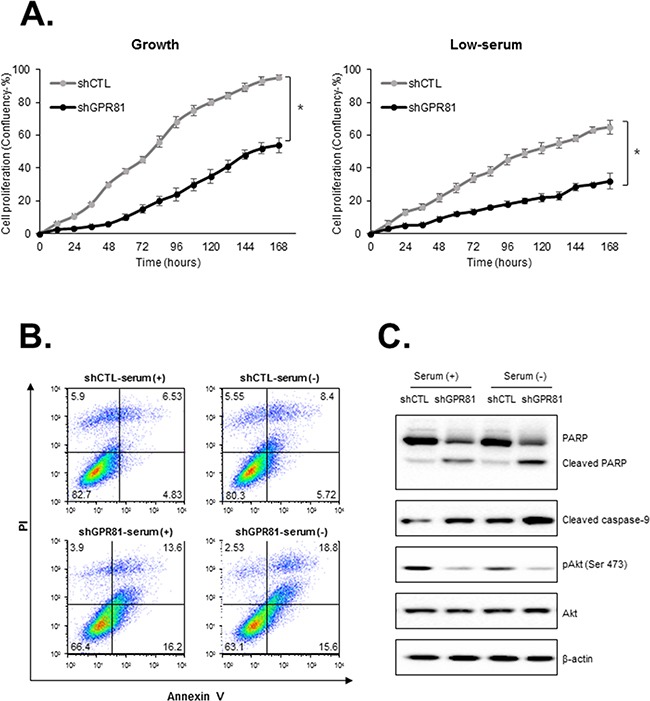
GPR81 knockdown impairs cell proliferation and increases apoptosis **A.** MCF7-shCTL or shGPR81 cells were seeded in 96-well plates and cultured under growth (10% serum, left) or low-serum (2% serum, right) conditions for 7 days. These data were condensed into quantifiable metrics using algorithms to generate a kinetic proliferation curve. The data are expressed as the mean±SEM; n≥3. *, *P* < 0.05 for the difference between cells that expressed control shRNA and cells with GPR81 shRNA. **B.** Flow cytometry (FACS) analysis of apoptosis in MCF7-shCTL and MCF7-shGPR81 cells. Early apoptosis, Annexin V^+^/propidium iodide (PI)^−^cells; late apoptosis, Annexin V^+^/PI^+^cells. **C.** Immunoblot analysis of Akt phosphorylation, PARP and caspase-9 cleavage at 24 h in the indicated conditions.

To investigate the physiological role of GPR81 in breast cancer progression, we generated stable knockdown cell lines (MCF7-shGPR81 and MCF7-shCTL) using lentiviruses that harbored shRNA. Two different shRNA sequences significantly reduced the GPR81 expression compared with MCF7-shCTL cells at the mRNA and protein levels (Supplementary Figures S2A and S2C). To assess whether GPR81 plays a role in breast cancer cell proliferation, we subsequently monitored the cell proliferation rate for 7 days at the indicated conditions (Figure 2A). Proliferation was significantly reduced to less than half in MCF7-shGPR81 cells under growth and low-serum conditions (Figure 2A). To determine whether the requirement for GPR81 for growth was a general feature of breast cancer cell lines, we suppressed GPR81 expression in an additional cell line, T47D (Supplementary Figure S2B). The T47D-shGPR81 cell lines grew more slowly than those infected with control shRNA in both growth and low-serum conditions (Supplementary Figure S3). These findings indicate that GPR81 increases the proliferation of breast cancer cells.

We subsequently investigated whether the GPR81 knockdown-induced loss of breast cancer viability resulted from apoptosis. As shown in Figure 2B, GPR81 knockdown in MCF7 cells led to an increased percentage of apoptosis (from 11.3% to 29.8%, Annexin-V (+) cells) under the growth condition (Figure 2B). Akt functions as a central regulator of cell survival by inhibiting apoptosis and promoting cell survival [19, 20]. Consistently, the Akt phosphorylation level was significantly decreased in MCF7-shGPR81 cells and increased the cleavage of poly (ADP-ribose) polymerase (PARP) and caspase-9, markers of apoptosis, in MCF7-GPR81 cells (Figure 2C). The opposite results were identified when the addition of the pan-caspase inhibitor Z-VAD-FMK enhanced the tumor cell growth exhibited in GPR81-knockdown cells (Supplementary Figure S4). Taken together, our findings indicate that the GPR81/Akt pathway is critical for breast cancer cell survival and apoptosis.

### GPR81 promotes breast cancer cell migration and invasion

To investigate the role of GPR81 in breast cancer metastasis, we measured the migratory and invasive capacity of GPR81-knockdown MCF7 cells. Using a Transwell assay, we demonstrated that the migration of GPR81-knockdown cells was reduced by 50% compared with control cells (Supplementary Figure S5A). In addition, using a Matrigel-coated Transwell invasion assay, we determined that the number of MCF7-shGPR81 cells that invaded through the Matrigel was significantly decreased compared with control cells (Supplementary Figure S5B). These findings indicated that GPR81 functions as a regulatory factor that promotes breast cancer cell aggressiveness.

### GPR81 activation promotes angiogenesis *in vitro*

**Figure 3:**
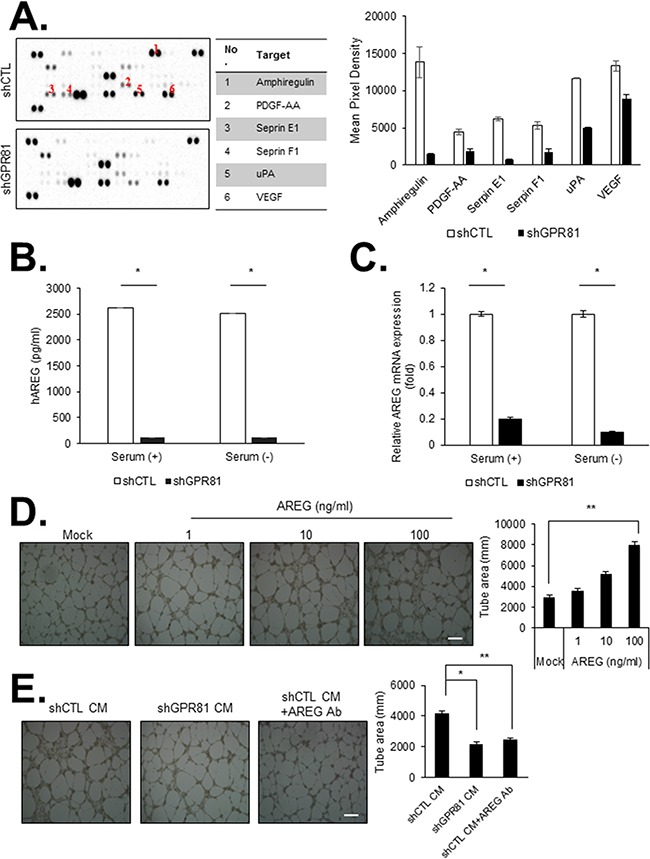
GPR81 activation promotes angiogenesis in primary human endothelial cells *in vitro* **A.** Angiogenic factor array of the conditioned media (CM) from MCF7-shCTL (top) and MCF7-shGPR81 cells (bottom) (left). The relative signal intensity of the indicated factor is presented (right). The data are expressed as the mean±SEM; n≥3. **B.** AREG levels in the CM from MCF7-shCTL and MCF7-shGPR81 cells were measured via an enzyme-linked immunosorbent assay (ELISA). *, *P*<0.005. The data are expressed as the mean±SEM; n≥3. **C.** AREG mRNA expression was measured via quantitative real-time PCR. All data are shown as the mean±SEM; n≥3; *, *P*<0.005. **D.** Representative images of HUVEC tube formation. HUVECs were treated with recombinant AREG at different concentrations. The bars represent the mean value of the tube area±SEM from five independent experiments (right). Scale bar, 20 μm; **, *P*<0.01. **E.** Representative images of HUVEC tube formation in the indicated conditions. Tube formation was quantified in five randomly selected fields (right). Scale bar, 20 μm; *, *P*<0.01; **, *P*<0.05.

Angiogenesis plays an essential role in tumor growth and metastasis [21, 22]. Tumor cells stimulate angiogenesis via the secretion of several pro-angiogenic factors, which promote tumor survival and metastasis through autocrine or paracrine signaling pathways. Thus, we investigated the effects of GPR81 activation on the regulation of cytokine and angiogenic factor expression in cancer cells. Among a panel of factors related to angiogenesis six factors were significantly decreased in the conditioned media (CM) from MCF7-shGPR81 cells compared with MCF7-shCTL cells; these factors included amphiregulin (AREG), platelet-derived growth factor (PDGF-AA), serpin peptidase inhibitor clade E (Serpin E1), serpin peptidase inhibitor clade F (Serpin F1), plasminogen activator (urokinase, uPA) and vascular endothelial growth factor (VEGF; Figure 3A).

Among the 6 factors regulated by GPR81, the AREG levels exhibited the greatest difference between the MCF7-shCTL and MCF7-shGPR81 cells at both the protein and mRNA levels (Figures 3B and 3C). AREG has been reported to stimulate cell proliferation and mammary gland development by acting as a ligand for the epidermal growth factor receptor (EGFR) [23, 24]. We initially aimed to determine whether tumor-derived AREG regulated the function of endothelial cells. We found that AREG promoted endothelial cell proliferation and migration in a dose-dependent manner (Supplementary Figures S6A and S6B). Furthermore, AREG dose- and time-dependently induced EGFR, Akt and ERK1/2 MAPK phosphorylation (Supplementary Figures S6C and S6D). AREG significantly induced *in vitro* endothelial cell (HUVECs) tube formation (Figure 3D) in a dose-dependent manner. To investigate the effect of tumor-derived AREG on endothelial cells, we incubated HUVECs with CM obtained from MCF7-shCTL cells and MCF7-shGPR81 cells. Consistently, the cells treated with the CM obtained from MCF7-shCTL cells showed efficient endothelial cell tube formation, whereas cells treated with the CM obtained from the MCF7-shGPR81 cells showed significantly impaired endothelial cell tube formation (Figure 3E). Strikingly, CM-induced tube formation was significantly decreased by neutralizing AREG with an anti-AREG antibody (Figure 3E). These findings indicate that GPR81 stimulates AREG production in MCF7 cells and promotes tumor angiogenesis in a paracrine loop. To determine the effects of AREG signaling on breast cancer cells, we subsequently treated MCF7-shCTL and MCF7-shGPR81 cells with AREG. AREG promoted cell proliferation and migration in a dose-dependent manner in the MCF7-shCTL and MCF7-shGPR81 cell lines (Supplementary Figures S7A and S7B). Consistently, the phosphorylated-EGFR and -Akt levels were slightly restored in AREG-stimulated MCF7-shGPR81 cells (Supplementary Figure S7C). Taken together, these findings suggest that the GPR81-induced AREG autocrine and paracrine loop plays a critical role in breast cancer progression.

### GPR81 signaling induces angiogenesis via PI3K/Akt pathway activation

**Figure 4:**
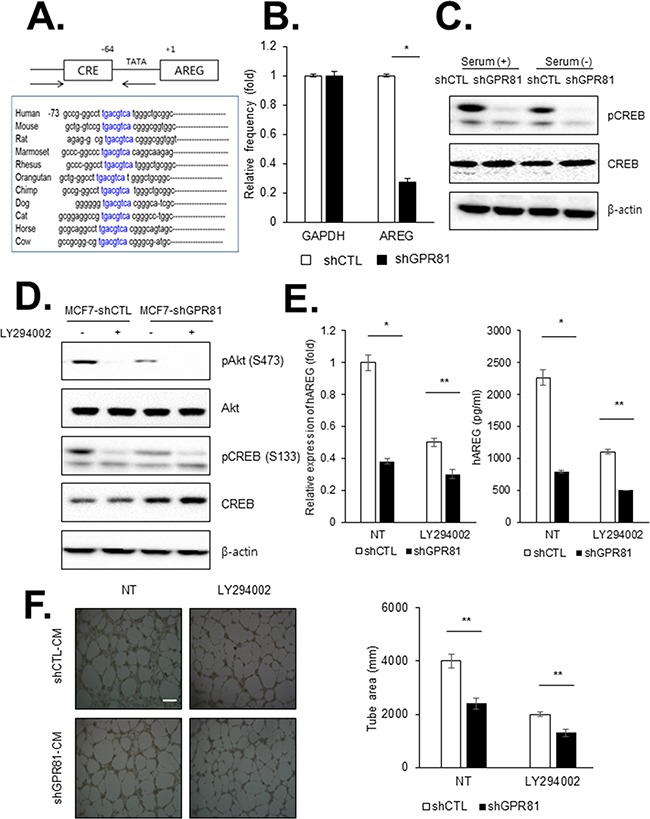
GPR81 signaling promotes angiogenesis via PI3K/Akt-CREB pathway activation **A.** CREB binding sites were enriched in the AREG promoter region. **B.** ChIP assay was performed with MCF7-shCTL and MCF7-shGPR81 cells using an anti-CREB antibody. AREG transcript levels were measured via qPCR analysis (*, *P*<0.01). The mean±SEM of three independent experiments is shown. **C.** MCF7-shCTL or MCF7-shGPR81 cells were seeded in 6-well plates and were treated with or without serum. CREB phosphorylation levels were measured via western blotting. **D.** Western blot analysis of cell lysates from MCF7-shCTL and MCF7-shGPR81 cells was used to detect phosphorylation of PI3K pathway components. β-actin was used as a loading control. **E.** AREG mRNA expression and protein secretion were measured via qPCR and ELISA assays, respectively. The data are expressed as the mean±SEM of three independent experiments; *, *P*<0.05;**, *P*<0.01. **F.** Representative images of HUVEC tube formation (left). HUVECs were treated with MCF7-shCTL CM and MCF7-shGPR81 CM with or without LY294002. Scale bar, 20 μm. Tube formation was quantified in five randomly selected fields. The bars represent the mean values of the tube area±SEM of five independent experiments. **, *P*<0.05 (right).

To define the molecular mechanism by which GPR81 modulates AREG transcription, we analyzed the transcriptional regulatory elements in the AREG promoter region. The CREB binding site is highly conserved in AREG orthologues from several different species (Figure 4A), and AREG may be regulated by CREB activation. To establish AREG as a specific CREB target gene, we performed chromatin immunoprecipitation (ChIP) using an anti-CREB antibody and primer designed to detect the promoter of AREG. Compared with control cells, the baseline binding of CREB to AREG promoters was reduced in GPR81-knockdown cells (Figure 4B). Consistent with this result, CREB phosphorylation was significantly reduced in MCF7-shGPR81 cells (Figure 4C). Therefore, the GPR81-mediated signaling pathway directly activates the AREG promoter via the transcription factor CREB.

CREB is a transcription factor activated by a diverse extracellular signals [25]. The well-established mechanism of CREB activation is the cAMP/protein kinase A (PKA) pathway. To determine whether PKA is the upstream molecule responsible for CREB activation, we measured the cAMP levels in MCF7-shCTL and MCF7-shGPR81 cells. However, we did not identify a significant difference in cAMP production between the MCF7-shCTL and MCF7-shGPR81 cells (data not shown). Therefore, we hypothesized that GPR81-induced CREB activation is regulated by another signaling pathway.

CREB activation may also be activated by PI3K/Akt; thus, our next step was to determine whether PI3K/Akt signaling was involved in GPR81-induced activation of CREB. As shown in Figure 4D, the basal phosphorylation of Akt and CREB were increased in MCF7-shCTL cells, whereas GPR81 depletion by shRNA blocked the activation of Akt and CREB. Consistent with this result, the treatment of LY294002, a PI3K inhibitor, potently reduced Akt and CREB activation both in MCF7-shCTL and MCF7-shGPR81 cells (Figure 4D). Moreover, LY294002 treatment inhibited the induction of AREG in MCF7-shCTL and MCF7-shGPR81 cells at both the mRNA and protein levels (Figure 4E). We subsequently performed a tube formation assay to determine whether GPR81-mediated angiogenesis was dependent on the PI3K/Akt-CREB pathway. As a result, GPR81-induced angiogenesis was inhibited in the presence of LY294002 (Figure 4F). Taken together, our findings suggest that angiogenesis enhanced by GPR81 signaling is mediated by the PI3K/Akt-CREB pathway via the regulation of angiogenic factors.

### GPR81 promotes orthotopic breast tumor growth and angiogenesis

**Figure 5:**
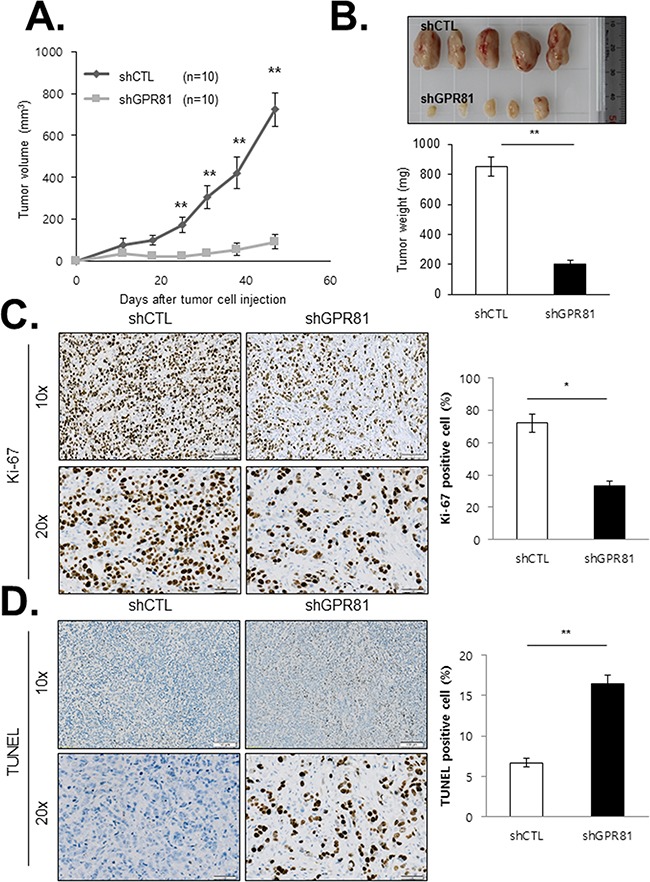
GPR81 depletion blocks breast cancer growth and survival in xenograft tumors **A.** Tumor volumes of MCF7-shCTL (n=10) and MCF7-shGPR81 (n=10) xenografts were measured over a period of 50 days after cell inoculation. The data are expressed as the mean volume±SEM; **, *P*<0.05. **B.** Representative images of the xenograft tumors (top) and tumor weights (bottom) at 50 days after tumor cell injection. The data are expressed as the mean weight±SEM; **, *P*<0.05. **C.** Ki-67-positive cell numbers were determined in individual xenograft tumors. Representative IHC staining of the tumors from each group of mice is presented (left). The bars represent the mean percentage of Ki-67-positive cells±SEM; *, *P*<0.01 compared with MCF7-shCTL mice (right). **D.** Representative images of TUNEL staining in tumor tissues from xenografts (left). The bars represent the mean percentage of TUNEL-positive cells (apoptotic cells)±SEM; **, *P*<0.05 compared with MCF7-shCTL mice (right).

To determine the effect of GPR81 signaling on breast cancer growth and angiogenesis, we generated an orthotopic xenograft mouse model. Two populations of MCF7 cells (MCF7-shCTL and MCF7-shGPR81) were orthotopically injected into the mammary fat pads of athymic nude mice, and tumor growth was monitored. As expected, the primary tumors from MCF7-shGPR81 mice grew at a significantly slower rate and were smaller in size compared with the MCF7-shCTL animals (Figures 5A and 5B). Consistently, histological examination indicated that the proliferation rates (Ki-67 labeling index) of the tumors from the mice injected with MCF7-shGPR81 cells were substantially decreased compared to those of tumors from mice injected with the MCF7-shCTL cells (Figure 5C). In addition, the proportion of apoptotic cells was increased in the tumor region of the MCF7-shGPR81-injected mice (Figure 5D). Collectively, these findings suggest that GPR81 is critical in modulating survival and apoptosis in breast cancer cells *in vivo*.

**Figure 6:**
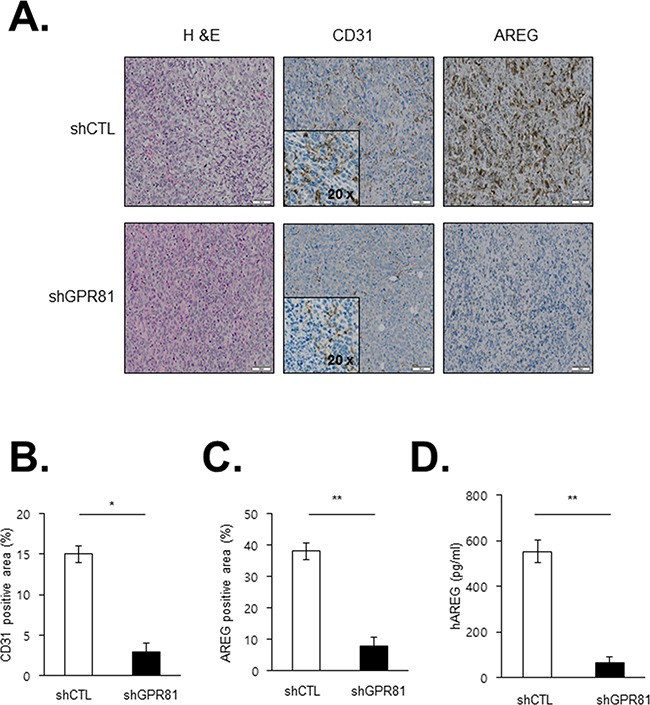
Effects of GPR81 silencing on angiogenesis in an orthotopic breast cancer model **A.** Representative microscopic images of tumor sections stained with H&E (left), CD31 (middle), and AREG (right) are presented. The scale bar represents 50 μm. **B.** Blood vessels (CD31-positive area) were quantified in tumor sections from xenografts. The data are expressed as the mean±SEM; *, *P*<0.05. **C.** AREG expression was quantified in tumor sections from the MCF7-shCTL and MCF7-shGPR81 mice. The data are expressed as the mean±SEM; **, *P*<0.05. **D.** Levels of human AREG in the blood obtained from the xenograft model. The data are expressed as the mean±SEM **, *P*<0.01.

**Figure 7:**
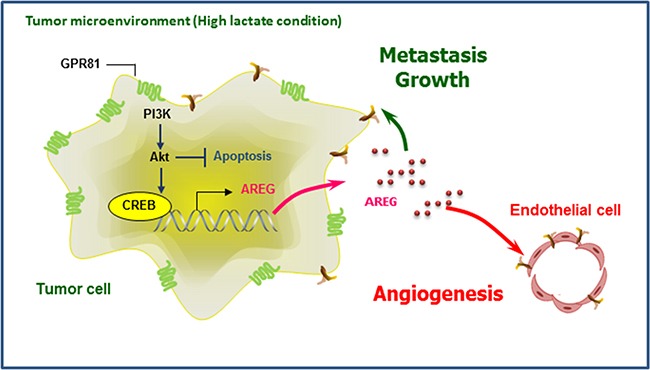
Schematic overview of GPR81 regulation of breast cancer progression During breast cancer progression, upregulated GPR81 is activated by extracellular lactate, which leads to PI3K/Akt pathway stimulation and the regulation of cell survival. Activation of the PI3K/Akt-CREB pathway results in increased AREG production, which AREG promotes neo-vessel formation (angiogenesis) in tumor endothelial cells and also provides a proliferative advantage to cancer cells and tumor growth.

We subsequently aimed to investigate the angiogenic effects of GPR81 *in vivo*. Compared with MCF7-shCTL-injected mice, MCF7-shGPR81-injected mice exhibited substantial reductions in microvascular density (CD31 positivity; Figures 6A, middle bottom and 6B), which indicates that GPR81 signaling promoted new blood vessel formation. Moreover, the AREG expression was significantly decreased in MCF7-shGPR81 tumors (Figures 6A, right bottom and 6C), and the AREG protein levels were also decreased in the serum of the MCF7-shGPR81 mice (Figure 6D). Taken together, these findings strongly suggest that GPR81 signaling enhances breast tumor growth *in vivo* likely through the inhibition of apoptosis and enhanced angiogenesis via AREG expression stimulation (Figure 7).

## DISCUSSION

In this study, we report a previously unknown role for GPR81 in breast cancer progression. In the tumor microenvironment, increased lactate levels significantly activated GPR81, which leads to the activation of cell survival signaling and the production of the angiogenic factor AREG to promote angiogenesis and cancer cell growth via PI3K/Akt pathway. Our results indicate that GPR81 is a putative tumor-promoting gene that promotes angiogenesis and the survival of breast cancer cells in tumor microenvironment.

Endogenous GPR81 expression has been identified in the adipocytes of humans and mice; however, it is also present at low levels in various normal cells such as skeletal muscle and liver [26, 27]. Here, we demonstrated that GPR81 was expressed in the normal human mammary epithelium at very low levels. Strikingly, we identified significantly increased GPR81 expression in a substantial cohort of human breast cancer tissues [28] and a panel of breast cancer cell lines. In addition, the GPR81 expression levels significantly correlated with the clinical characteristics of breast cancer, particularly ER status (Supplementary Figure S1B). This is consistent with our observation that ER-positive breast cancer cells (MCF7 and T47D) expressed GPR81 at a higher level (Figure 1A). Recent studies have identified GPR81 expression in several cancer cell types, including colon, lung and breast cancers [29, 30], which is in agreement with the GPR81 expression pattern identified in the current study (Figures 1A-1C). These findings suggest that GPR81 may be a prognostic marker and GPR81-mediated signaling could play a key role in the progression of breast cancer.

Altered cellular metabolism is a hallmark of cancer (the Warburg effect) and the lactate concentrations range from 5 to 20 mmol/L in the tumor microenvironment [31]. Although lactate has generally been considered as a by-product of metabolism, recent evidence suggest that lactate functions as an active metabolite and stimulates signaling pathway [32, 33]. We investigated whether lactate is involved in GPR81-mediated signaling pathway in high lactate concentration. Exogenous lactate did not stimulate the cell viability of MCF7-shCTL or MCF7-shGPR81 cells (Supplementary Figure S8A). Moreover, exogenous lactate had no effect on the Akt phosphorylation of either MCF7-shCTL and MCF7-shGPR81 cells (Supplementary Figure S8C). However, the growth of MCF10A cells transfected with myc-tagged GPR81 more increased than those of cells transfected with empty vector (Supplementary Figure S8B). Furthermore, exogenous lactate slightly increased the cell viability of MCF10A-myc-GPR81 cells. Therefore, breast cancer cells were not sensitive to exogenous lactate as a ligand, and GPR81 may be constitutively activated in the high lactate concentration of tumor microenvironment.

The activation of GPR81 subsequently inhibits lipolysis and β-oxidation and, as recently demonstrated, increases the mRNA expression levels of genes critical for lactate metabolism [29]. These metabolic changes may partially influence cancer cell survival. However, increased lactate levels could act as a signal to activate its specific receptor, not as a metabolic fuel. In current study, the loss of GPR81 in MCF7 cells led to a loss of viability and increased apoptosis *in vitro* and *in vivo.* One well-established pathway, the PI3K/Akt pathway, mediates pro-survival signals in various types of cancers [20]. In particular, Akt is involved in the inhibition of apoptosis via the phosphorylation of pro-apoptotic molecules, e.g., Bad or caspase-9, or the modulation of transcription factors, such as c-Raf [34]. At the molecular level, constitutive Akt activation (phosphor-Akt) was suppressed, and PARP cleavage and caspase-9 activity were increased in MCF7-shGPR81 cells, indicating an apoptotic signature. GPR81 knockdown-induced apoptosis was reversed, in part, by treatment with a pan-caspase inhibitor (Supplementary Figure S4). Thus, PI3K/Akt inhibition-induced caspase-dependent apoptosis appears to comprise a key factor in the GPR81-mediated cell survival pathway.

Angiogenesis, the process of neo-vessels formation, has a key role in breast cancer growth and progression [35]. Angiogenic switch, the shift in the balance between proangiogenic and antiangiogenic factors in favor of pro-angiogenesis, applies to almost types of solid tumors [36, 37]. Here, we found that AREG is a critical factor in GPCR-induced angiogenesis. AREG is a ligand of EGFR [38] that plays a central role in mammary gland development and branching morphogenesis in organs [39]. AREG plays a central role in numerous physiological and pathological processes, especially in cancer progression and development [40]. Here, we showed that GPR81 signaling enhanced angiogenesis via the induction of several genes involved in angiogenesis including AREG *in vitro* and *in vivo*. Furthermore, we demonstrated cancer-derived AREG partially promoted breast cancer growth and migration via an autocrine AREG/EGFR signaling pathway (Supplementary Figures S7). These findings suggested that GPR81 signaling-induced AREG promoted tumor cells to switch to an angiogenic phenotype, thereby leading to tumor progression in an autocrine/paracrine manner.

Our results also revealed the molecular mechanisms that underlie GPR81-induced angiogenesis. CREB plays important roles in the development of malignant phenotypes, and its aberrant activation has been demonstrated in various cancer cell types [41]. Numerous proangiogenic genes, such as VEGF and endothelin-1, are transcriptionally regulated by the CREB pathway [42, 43]. We demonstrated that GPR81 activation promoted CREB phosphorylation in MCF7 cells, which is essential for the translocation of active CREB to the nucleus. Furthermore, blocking CREB activation completely abrogated the GPR81 signaling-induced production of AREG and angiogenesis. CREB activation is mediated by several upstream signaling pathways [41]. cAMP-dependent protein kinase phosphorylated CREB is a well-known signaling pathway; however, GPR81 activation did not change the concentration of cAMP in our cell system (data not shown). In contrast, our data suggested the PI3K/Akt pathway was indeed activated by GPR81. The suppression of PI3K/Akt signaling abrogated the GPR81-induced effects on CREB activation and subsequently inhibited AREG expression and angiogenesis. These data support that the PI3K/Akt-CREB pathway contributes to the tumor-associated angiogenesis mediated by aberrant GPCR signaling in tumor microenvironment.

In conclusion, our current results show that GPR81 functions as a tumor-promoting receptor that senses extracellular lactate in human breast cancer. GPR81 signaling activation promotes cell survival and angiogenesis, mainly by stimulating Akt activation and proangiogenic factor production, respectively. Therefore, our findings provide extended mechanistic clues that contribute to a better understanding of both the physiological roles of GPR81 and its potential as an alternative anti-angiogenic therapeutic target in cancer.

## MATERIALS AND METHODS

### Cell culture

The breast cancer cell lines MCF7, T47D, MDA-MB-231, SK-BR-3, MDA-MB-453, MDA-MB-468, Hs578T and MCF10A were acquired from the American Type Culture Collection (ATCC, Manassas, VA, USA), where they are regularly authenticated. The cell lines were grown at 37°C in 5% CO_2_. Hs578T, MDA-MB-231, MDA-MB-453, MDA-MB-468, and SK-BR-3 cells were maintained in DMEM (Lonza, Basel, Switzerland) containing 10% fetal bovine serum (FBS, Gibco BRL, Grand Island, NY, USA), penicillin (100 units/ml; Gibco) and streptomycin (100 units/ml, Gibco). MCF7 and T47D cells were cultured in RPMI 1640 (Lonza), 10% FBS, penicillin (100 units/ml) and streptomycin (100 units/ml). MCF10A cells were cultured in DMEM/F12 media (1:1) (Invitrogen, Grand Island, NY, USA) supplemented with 5% horse serum (Gibco), 10 μg/ml bovine insulin (Sigma-Aldrich, St. Louis, MO, USA), 20 ng/ml epidermal growth factor (EGF; Sigma), 0.5 μg/ml hydrocortisone (Sigma), 0.1 μg/ml cholera toxin (Sigma), penicillin (100 units/ml), and streptomycin (100 units/ml). Human umbilical vein endothelial cells (HUVECs; Lonza) were maintained in Lonza EGM-MV (normal growth medium) at 37°C in 5% CO_2_. The cells were maintained in culture plates and used in assays between cell passages 3 and 8.

### Clinical specimens

The primary tumors analyzed in this study were obtained from the Seoul National University College of Medicine in compliance with the policies and practices of the SNU Internal Review Board. Tumor-adjacent normal breast tissue from 20 patients undergoing breast reduction surgery and benign or aggressive breast tissue samples from 60 patients were collected at the SNU hospital. Immediately after biopsy, the tissue samples were frozen in liquid nitrogen and stored at −70°C until use.

### Mammalian lentiviral shRNAs

MCF7 and T47D cells were infected with a recombinant non-replicative lentiviral plasmid (Sigma, St. Louis, MO, USA) containing human shGPR81 (transfected with 2 different shRNA constructs for GPR81) or with a control plasmid (pLKO.1-puro) obtained from Sigma. Each construct was co-transfected with the packaging constructs VSVG (viral glycoprotein expression vector) and delta 8.9 (packaging vector) packaging constructs. Lentivirus was produced in 293T cells using the LipofectAMINE 2000 reagent (Invitrogen, Carlsbad, CA, USA). The medium was replaced at 16 h after transfection, and the supernatant was harvested after an additional 48 h. The lentiviral particles were used to transduce the target cells for 24 h. The cells were infected with lentivirus (500 μl of supernatant/ml medium) mixed with polybrene (4 μg/ml). Puromycin-resistant clones (3 μg/ml) were then isolated using the limiting dilution method. GPR81 knockdown was verified by RT-PCR and Immunocytochemistry.

### Cell proliferation assay

An IncuCyte™ Live-cell Imaging System (Essen BioScience, Ann Arbor, MI, USA) was used to monitor the kinetics of cell growth, as determined by the assessment of cell confluency under indicated conditions. Additionally, time-lapse phase-contrast images were automatically collected once per hour from cells grown for 7 days in CO_2_ incubator. Cell growth was quantified and is shown as monolayer confluence versus time.

### Detection of tumor-derived angiogenic factors using antibody arrays

Human angiogenic protein array kit (R&D Systems) was used according to the manufacturer's instructions. Briefly, the membranes were blocked, incubated with 100 μl of conditioned media (CM) overnight, and incubated with biotin-conjugated antibodies (1/250) for 2 h and with an HRP-linked secondary antibody (1/1000) 30min. Next, the membranes were incubated with a chemiluminescent substrate and exposed. The reactive proteins were visualized and analyzed using an ECL Kit (Thermo Scientific, Rockford, IL, USA) and ImageQuant™ LAS 4000 (GE Healthcare, Buckinghamshire, UK). Quantitative array analysis was performed using Image J software.

### Tube formation

Pooled HUVECs were purchased from Lonza and cultured according to the supplier's instructions. For the Matrigel tube-formation assay, reduced growth factor Matrigel™ (BD Biosciences, Bedford, MA, USA) was thawed overnight at 4°C. The Matrigel was allowed to solidify in 48-well culture dishes at 37°C for 30 min. The cells were harvested and seeded at a density of 2×10^5^ cells/well in the presence or absence of recombinant AREG. In separate experiments, the cells were either treated with an AREG-neutralizing antibody (R&D Systems, Minneapolis, MN) or conditioned medium collected from MCF7-shCTL and MCF7-shGPR81 cells. The cells were then incubated at 37°C for an additional 12 h. Tube formation was observed by capturing images using an Olympus CellR microscope. The Matrigel assay results were quantified by measuring the total number of pixels in thresholded images using MetaMorph software (Molecular Devices, Sunnyvale, CA, USA). Three independent experiments were performed, and each experiment was performed in triplicate. Student's *t*-test was performed to determine the significance between the test groups.

### Chromatin immunoprecipitation (ChIP) analysis

MCF7-shCTL and MCF7-shGPR81 cells processed for ChIP as described previously [44]. Briefly, collected cells were treated with 1ml Lysis buffer with protease inhibitors (ChIP kit, EMD Millipore, Billerica, MA, USA) and were sonicated using the Misonix Sonicator 3000 (Newton, CT, USA) at high power until DNA fragments of 200-1000bp were formed. The sonicated chromatin was precipitated using anti-CREB antibody (Cell signaling Technology) or rabbit IgG antibody (negative control). Precipitated complexes were eluted and treated with Proteinase K (100 μg/ml) and DNA fragments were purified by Qiagen Quick kit (Qiagen, Hilden Germany). The DNA input and comparison between MCF7-shCTL and MCF7-shGPR81 cell line as control were used for qPCR. The PCR primer of AREG promoter region and GAPDH control are listed below (forward, reverse):

Human GAPDH: 5'-AAAAGCGGGAGAAAG TAGG-3', 5'-CTAGCCTCCCGGGTTTCTCT-3'

Human AREG: 5'-TTTTCGGGTAGCACCTTC TG-3', 5'-CAGGTGTGCGAACGTCTGTA-3'

Data was normalized to input DNAs. Differential Ct (cycle threshold) values from experimental and input DNAs (ΔCt) were used to calculate amplified DNA yield.

### Animals and xenograft models

Animal handling and experimentation were performed with the approval of the Institutional Animal Care and Use Committee of the Ulsan National Institute of Science and Technology (UNIST). 6-8 week old athymic nu/nu mice (Harlan, Madison, WI, USA) were subcutaneously implanted with 17β-estradiol pellets (0.72 mg, 60-day release; Innovative Research of America, Sarasota, FL, USA) before being inoculated with the cells. A total of 5×10^6^ MCF7-shCTL or shGPR81 cells were inoculated into the mammary fat pads of the animals (10 per group). The tumor volume was measured using a digital slide caliper and was defined as follows: (1/2)×(long diameter)×(short diameter)^2^.

### Statistical analyses

Data were analyzed using GraphPad software (GraphPad Prism version 7.0 for Windows). All of the data measurements are presented as the mean ± SEM. Statistical significance was evaluated by the Student's *t*-test. Values of *P*<0.05 were considered to be statistically significant.

## SUPPLEMENTARY INFORMATION, SUPPLEMENTARY FIGURES


