# Supplementary material for: G-protein-coupled receptor 81 promotes a malignant phenotype in breast cancer through angiogenic factor secretion
Source: Oncotarget. 2016 Sep 27;7(43):70898–911. doi: 10.18632/oncotarget.12286 (PMC5342597; doi:10.18632/oncotarget.12286)
Supplement: Supplementary file 1 [file oncotarget-07-70898-s001.pdf]

G-protein-coupled receptor 81 promotes a malignant phenotype in breast cancer through angiogenic factor secretion

SUPPLEMENTARY INFORMATION

MATERIALS AND METHODS

Immunoblot analysis

Cells exposed to the indicated conditions were washed twice with ice-cold PBS and lysed with RIPA buffer (Thermo Scientific) containing protease and phosphatase inhibitors (Roche). Equal amounts of protein per lane were separated on a 6-16% gradient sodium dodecyl sulfate polyacrylamide gel electrophoresis (SDS-PAGE) gel and transferred to a nitrocellulose membrane. The membrane was blocked with 5% non-fat dry milk and probed with anti-p42/p44 MAPK, pEGFR Y1068, pAkt, EGFR (Cell Signaling Technology, Danvers, MA, USA), anti-ERK2, Akt, β-actin, and GFP antibodies (Santa Cruz Biotechnology, Santa Cruz, CA, USA) at 4°C overnight. The bound primary antibodies were visualized using horseradish peroxidase-conjugated secondary antibodies (Kirkegaard & Perry Laboratories, KPL, Gaithersburg, MD, USA) and an ECL system (Amersham, Uppsala, Sweden) with ImageQuant™ LAS 4000 (GE Healthcare, Buckinghamshire, UK).

RNA reverse transcription and quantitative PCR analysis

The total RNA was extracted from the cells with Trizol® Reagent (Invitrogen) according to the manufacturer's protocols. The extracted RNA was reverse transcribed into cDNA using SuperScript® II Reverse Transcriptase (Invitrogen). Quantitative reverse transcription PCR (qRT-PCR) was performed with a LightCycler 480 instrument (Roche Diagnostics, Switzerland) using Fast SYBRgreen master mix (Roche), according to the manufacturer's instructions. All reactions were performed in a 20-μl reaction volume in triplicate. The primers were obtained from Invitrogen. Following an initial denaturation at 95°C for 30 s, 40 cycles of PCR amplification were performed at 95°C for 5 s and 60°C for 20 s. Standard curves were generated and the relative amount of the target gene mRNA was normalized to that of GAPDH. Specificity was verified by a melting curve analysis. We used the comparative cycle threshold method to compute the relative expression values. Primers designed using online primer tools Primer3 are listed below (forward, reverse):

Human GPR81: 5'-CTGGTCATCCTGGGAACAGT-3', 5'-CTTCTTCATCCGAGCCTGTC-3'

Human AREG: 5'-GAGAAGCTGAGGAACGAAAGAA-3', 5'-AGGACCGACTCATCATTTATGG-3'

Human β-actin: 5'-AGAGCTACGAGCTGCCTGAC-3', 5'-AGCACTGTGTTGGCGTACAG-3'

Transwell migration

The assays were performed in 24-well Boyden chambers (Corning, NY, USA). Filters (8-μm pore size) pre-coated with collagen type I were used to examine cell invasion. MCF7 cells were detached from the plates, and the cell suspension was placed into the upper chamber in 0.5 ml of RPMI 1640 serum-free medium (1 x 10^5 cells per filter). RPMI 1640 media supplemented with 10%FBS or 0.2%FBS were placed in the lower chamber as chemoattractants. Migration were scored after 5-6 h. Cells on the lower surface of the filters were fixed in 4% PFA for 20 min at room temperature, stained using crystal violet for 5 min, visualized and counted. The values for cell migration and invasion were expressed as the mean number of cells per microscopic field in five fields per one filter in triplicate experiments. The experiments were repeated at least three times.

Histological analysis

For hematoxylin and eosin (H&E) staining, the tumors, and the indicated organs were fixed overnight in 10% formalin. After tissue processing using standard procedures, the samples were embedded in paraffin and cut into 4-μm sections, followed by H&E staining. For immunohistochemistry analyses, the tissue blocks were cut into 4-μm sections. The samples were blocked with 5% goat serum in PBST (0.05% Triton X-100 in PBS) and then incubated for 3 h at room temperature (RT) with the following primary antibodies: anti-Ki-67 (Dako, Carpinteria, CA, USA), anti-CD31 (BD Pharmingen, San Diego, CA, USA); and anti-AREG (R&D Systems). Staining was performed according to the standard protocols. Apoptosis was determined by the terminal transferase-dUTP-nick end labeling (TUNEL) assay in primary tumor sections using an ApopTag in situ apoptosis detection kit (S7101, Chemicon International, Temecular, CA, USA). The slides were counterstained with hematoxylin, dehydrated, and mounted using a synthetic mounting medium (Dako) and observed using a digital virtual microscope (dotSlide; Olympus) at the UNIST-Olympus Biomed Imaging Center (UOBC) and analyzed by MetaMorph image software (Molecular Devices, Sunnyvale, CA, USA).

SUPPLEMENTARY FIGURES

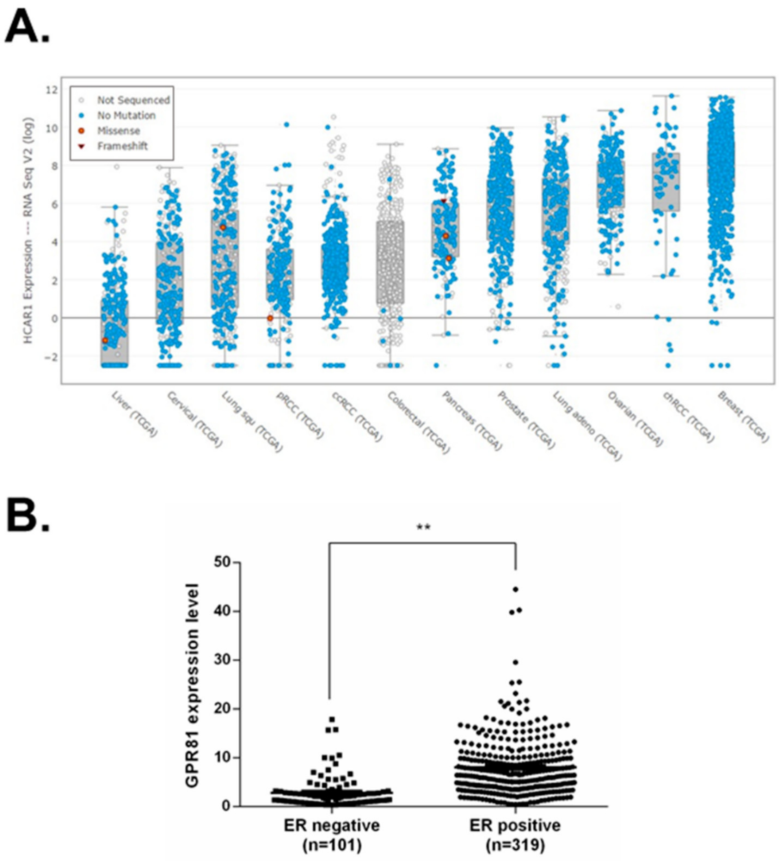

Supplementary Figure S1: GPR81 is highly expressed in breast cancer. A. Analysis of large-scale cancer genomic datasets reveals frequent GPR81 gene amplification in different cancer types. Data analysis and visualization were performed using the cBioPortal Cancer Genomics website (http://www.cbioportal.org). **B.** Gene expression analysis of GPR81 expression in 420 human breast tumors (TCGA cohort) stratified by estrogen receptor (ER) status (TCGA cohort, n= 420). **, *P*<0.0001.

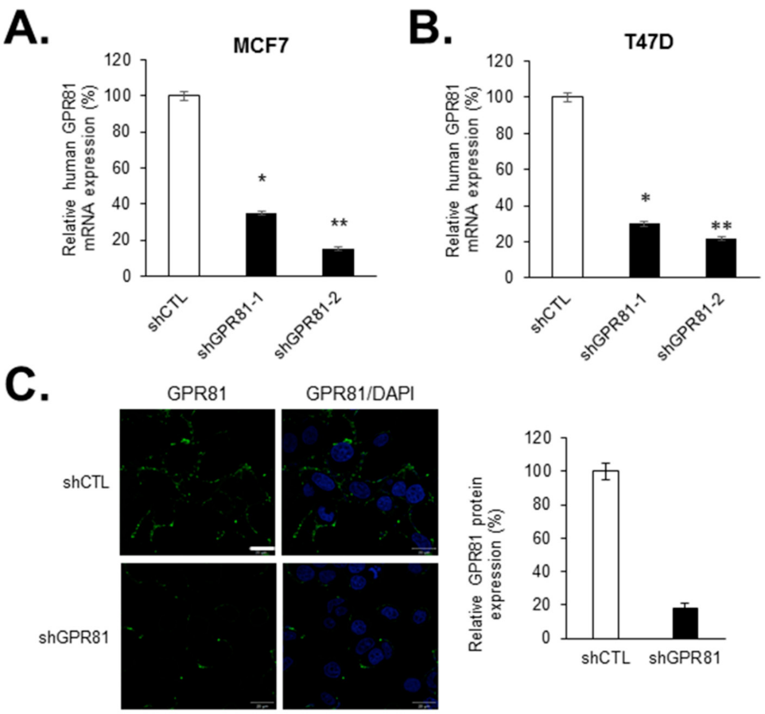

Supplementary Figure S2: GPR81 expression in stable GPR81 knockdown cell lines. A. MCF7 cells were transfected with scramble shRNA (shCTL) or GPR81 shRNA-expressing constructs (shGPR81-1 and -2). The shGPR81 cell lines exhibited a substantial reduction in the GPR81 mRNA levels. Results show the mean±SEM (n=3), normalized to shCTL. *, $P<0.01$; **, $P<0.001$. **B.** Relative mRNA expression of GPR81 in T47D cells following lentiviral stable knockdown of GPR81 (shGPR81) or lentiviral control (shCTL). Data represent the mean±SEM (n=3). **C.** MCF7-shCTL and MCF7-shGPR81 cells were immunostained for GPR81 (green) and DAPI (blue) (left). Scale bar, 20 μm. Quantification of GPR81 protein levels (right). Data represent the mean±SEM (n=3).

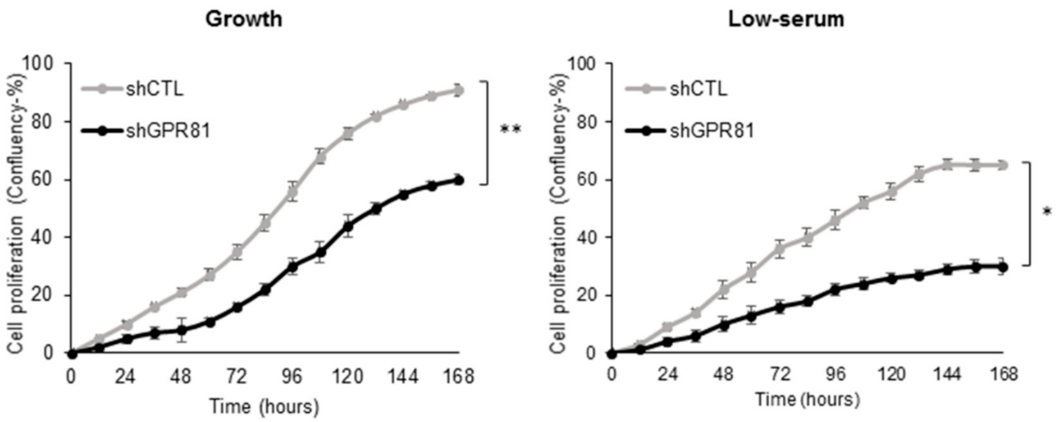

Supplementary Figure S3: shRNA-mediated knockdown of GPR81 strongly attenuates breast cancer cell growth *in vitro*. Cell proliferation of T47D cells transduced with shGPR81 constructs under growth (10% serum, left) or low-serum (2% serum, right) conditions. Results represent the mean±SEM (n=3). *, $P<0.01$; **, $P<0.001$.

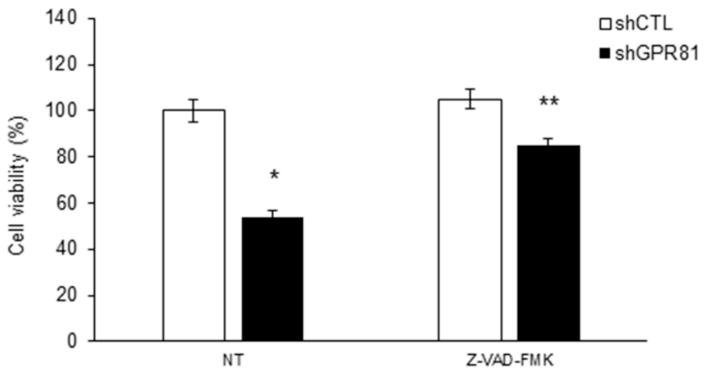

Supplementary Figure S4: Effect of an apoptotic inhibitor on GPR81-knockdown cells. Treatment with a pan-caspase inhibitor, Z-VAD-FMK, reverses the tumor cell death demonstrated in MCF7-shGPR81 cells. Data represent the mean±SEM (n=3). *, $p<0.05$ shGPR81 *versus* shCTL, **, $p<0.05$ shGPR81+Z-VAD-FMK *versus* shGPR81.

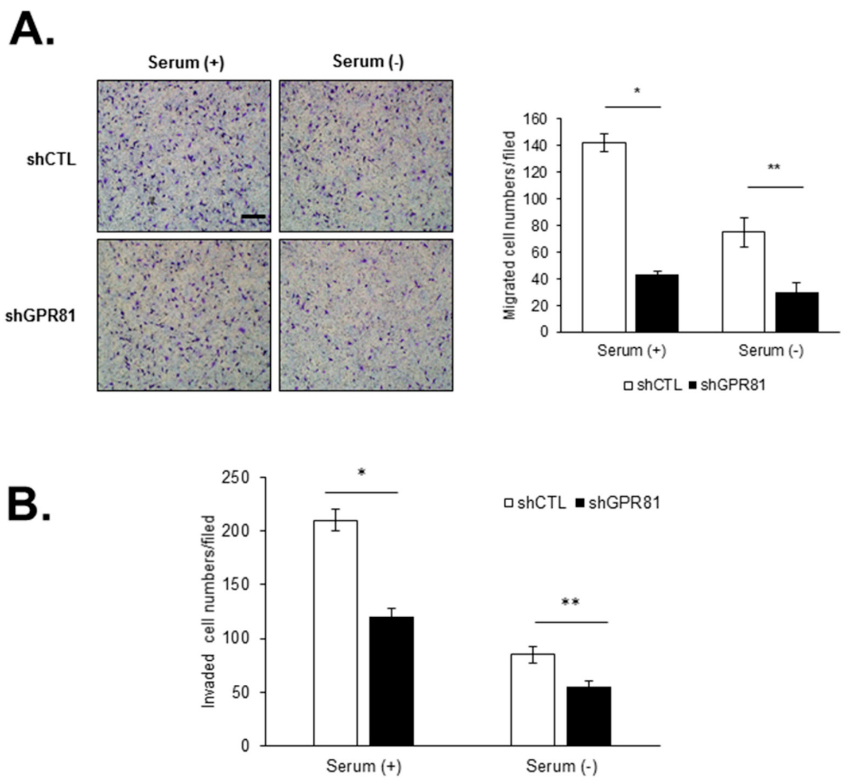

Supplementary Figure S5: shRNA-mediated knockdown of GPR81 strongly attenuates breast cancer cell migration and invasion *in vitro*. A. Cell migration was measured using Transwell assays for MCF7-shCTL and MCF7-shGPR81 cells. Crystal violet-stained migrated cells were counted with an inverted microscope. Scale bar, 50 µm. *, $P<0.01$; **, $P<0.05$. The data are expressed as the mean±SEM of five independent experiments for either Transwell migration assay. **B.** Cell invasion was measured using Transwell invasion assays for MCF7-shCTL and MCF7-shGPR81 cells. Data represent the mean±SEM (n=3). *, $P<0.05$; **, $P<0.001$.

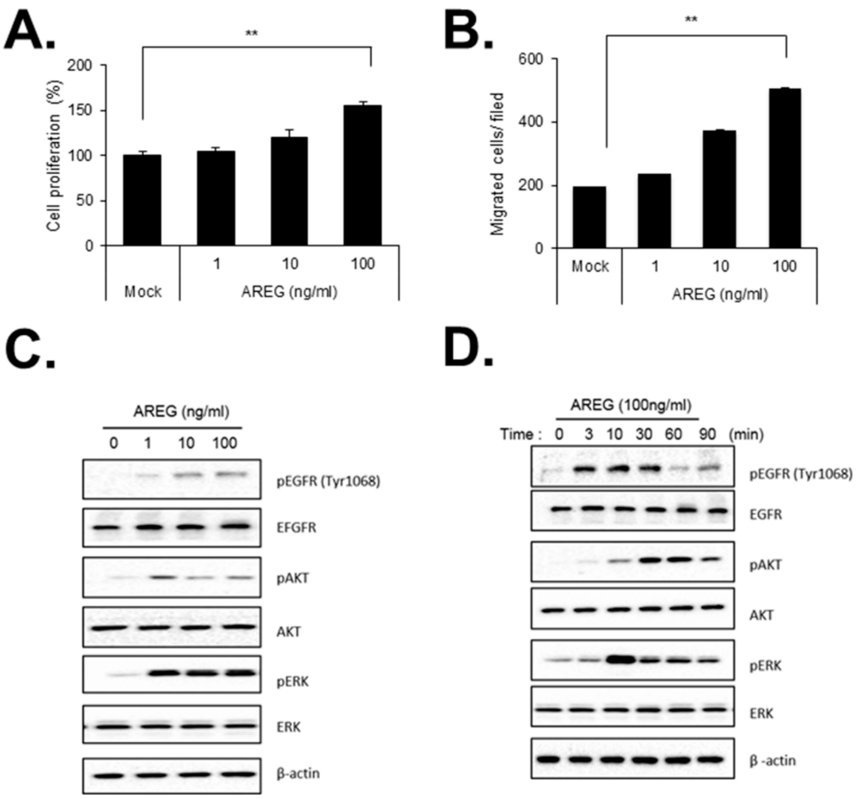

Supplementary Figure S6: Effects of AREG on human endothelial cell proliferation and migration. A. The MTT assay was used to measure cell proliferation in HUVEC cells at different AREG concentrations. **, *P*<0.05, compared with Mock treatments (mean±SEM, n=3). **B.** Transwell migration assay for HUVECs with or without AREG. **, *P*<0.01, compared with Mock treatments (mean±SEM, n=3). **C.** Western blotting for phosphorylated EGFR, Akt and ERK in HUVECs treated with or without AREG (dose-dependent). **D.** Western blotting for phosphorylated EGFR, Akt and ERK in HUVECs treated with or without AREG (time-dependent).

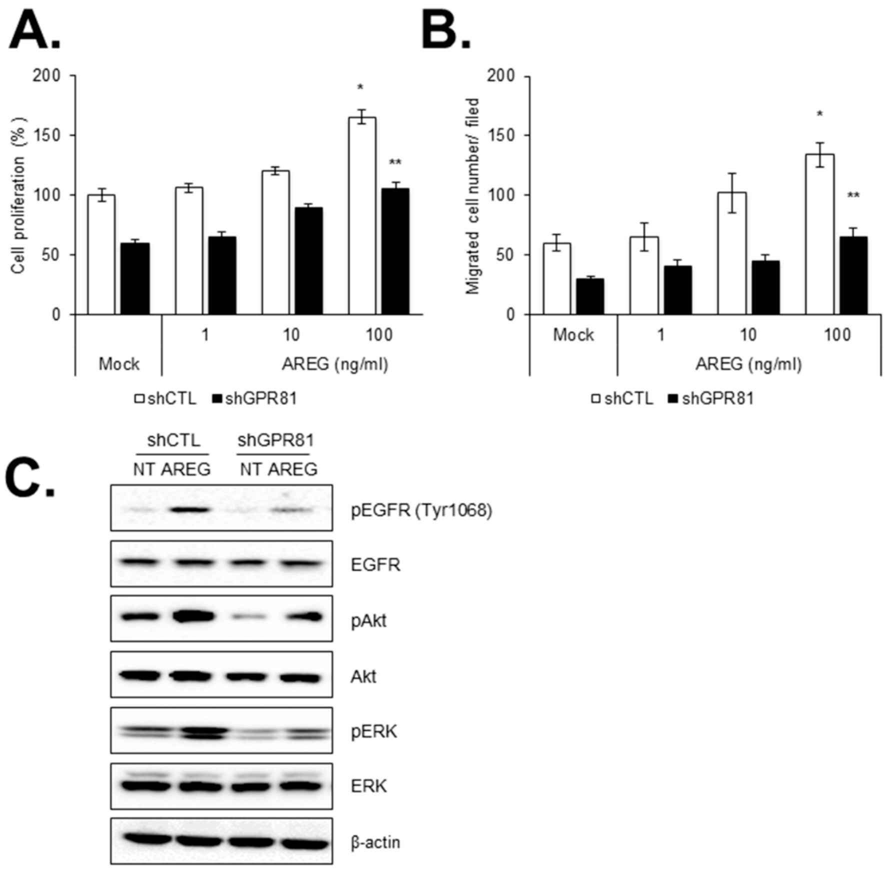

Supplementary Figure S7: Effects of AREG on breast cancer cell proliferation and migration. A. The MTT assay was used to measure cell proliferation in MCF7-shCTL and MCF7-shGPR81 cells at different AREG concentrations. **B.** Transwell migration assay for MCF7-shCTL and MCF7-shGPR81 cells under AREG treatment. *, *P*<0.01; **, *P*<0.05. (mean±SEM, n=3). **C.** MCF7-shCTL and MCF7-shGPR81 were seeded in 6-well plates and treated with AREG (100ng/ml) for 10 min. pEGFR, pAkt and pERK levels were measured via western blotting. The results are representative of three independent experiments.

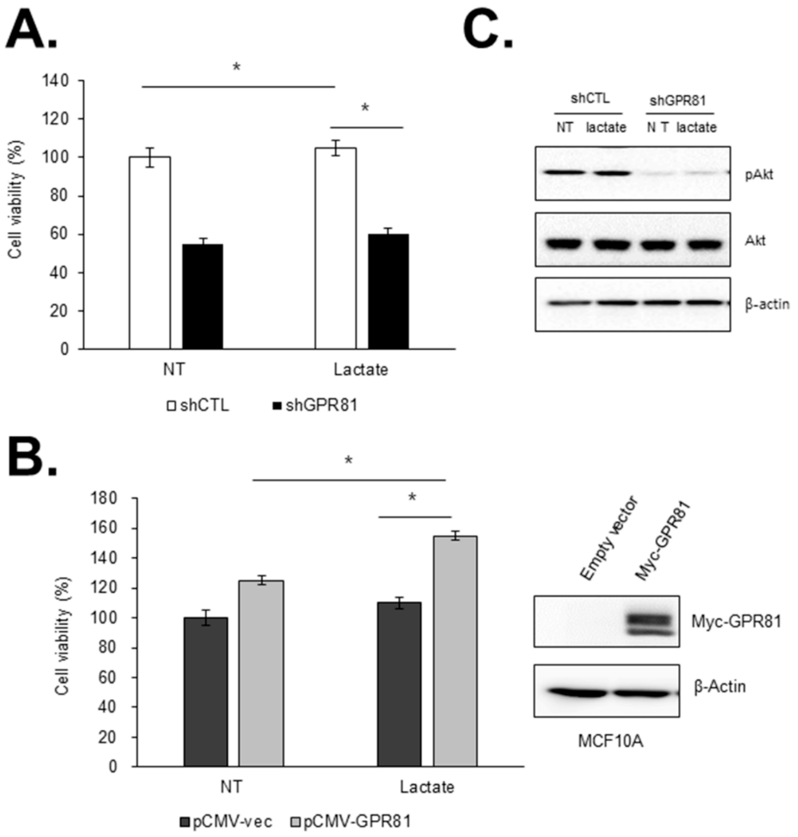

Supplementary Figure S8: Exogenous lactate does not activate the GPR81 downstream signaling pathway. A. Cell viability of MCF7-shCTL and MCF7-shGPR81 cells was measured with the MTT assay with or without exogenous lactate at 48 h. Results represent the mean±SEM (n=3), normalized to shCTL-NT. **B.** MCF10A cells transfected with myc-tagged GPR81 or empty vector were grown with or without lactate. Cell viability was determined by MTT assay. The representative immunoblot with anti-myc indicates GPR81 expression level. *, P<0.01 versus empty vector (NT). All values shown are mean±SEM (n=3). **C.** Western blot analysis of cell lysates from MCF7-shCTL and MCF7-shGPR81 cells was used to detect pAkt.
